# Parent and Therapist Perceptions of the Feasibility, Acceptability, and Benefits of a Weekly Therapist-Led Massage Program for Extremely Preterm Infants in Neonatal Intensive Care

**DOI:** 10.3390/children10091453

**Published:** 2023-08-25

**Authors:** Dana B. McCarty, Stacey C. Dusing, Alana Gilbert, Kristen D. LeBlond, Meredith Soucie, T. Michael O’Shea

**Affiliations:** 1 Department of Health Sciences, University of North Carolina at Chapel Hill School of Medicine, Chapel Hill, NC 27599, USA; 2 Rehabilitation Services, University of North Carolina Children’s Hospital, Chapel Hill, NC 27599, USA; 3 Division of Biokinesiology and Physical Therapy, University of Southern California, Los Angeles, CA 90033, USA; 4 Frank Porter Graham Child Development Institute, University of North Carolina at Chapel Hill, Chapel Hill, NC 27599, USA; 5 Duke Physical and Occupational Therapy, Duke Health, Chapel Hill, NC 27517, USA; 6 Department of Pediatrics, University of North Carolina at Chapel Hill School of Medicine; Chapel Hill, NC 27599, USA

**Keywords:** extremely preterm infants, neonatal intensive care unit, parent, mother, mental health, physical therapy, occupational therapy, infant massage

## Abstract

Mothers of extremely preterm infants experience high rates of mental health disorders that impair maternal–infant interaction and lead to worse infant developmental outcomes. Therapist Education and Massage for Parent–Infant Outcomes (TEMPO) is a therapist-led program that standardizes the nature and frequency of parent education through weekly scheduled therapy sessions. Using a family-centered approach, the therapist facilitates positive maternal–infant interactions and massage interventions from birth throughout hospitalization with the goal of improving maternal mental health. This qualitative study presents the results of 19 parent interviews and of a focus group of four TEMPO interventionists to elicit feedback about the program. Overall, parents and therapists viewed the program positively. Parents and therapists valued the focus on parent education and engagement to increase parent competence and bonding opportunities. Both groups acknowledged that infant massage had both infant-centered and parent-centered benefits. One area where parent and therapist views did not align was regarding feasibility of TEMPO. Parents noted multiple logistical challenges to regular NICU visitation, but ultimately agreed that attending weekly therapy sessions was feasible. Therapists noted increased time and effort required of TEMPO and felt that institutional and system-level changes would be necessary to implement weekly parent education as standard of care.

## 1. Introduction

Mothers of preterm infants are at high risk of developing mental health disorders. Up to three-fourths of these mothers experience anxiety [1], and their rates of postpartum depression are 40% higher than mothers of healthy infants [2]. Mothers of extremely preterm infants, born prior to 28 weeks gestational age (GA), are most likely to develop these symptoms [3] in part because such infants require prolonged medical intervention in the neonatal intensive care unit (NICU), contributing to maternal feelings of helplessness and stress [4] and creating a barrier to maternal–infant attachment [5]. Ultimately, maternal anxiety and depressive symptoms impair maternal–infant interaction, leading to worse developmental outcomes of the infant [1,5].

Higher maternal visitation rates and more maternal–infant physical contact (e.g., skin-to-skin, blanket holding) are associated with improved infant outcomes, maternal-child bonding, maternal health behavior, and maternal mental health [6,7]. In a meta-analysis of interventions to reduce parent stress and trauma in a hospital setting, complementary and alternative interventions, such as massage, reduced parent mental health symptoms [8]. Therefore, a multi-component intervention to support maternal visitation and maternal–infant physical contact through a modality like massage may improve multiple outcomes, including maternal mental health.

Physical therapy (PT) and occupational therapy (OT) for neonates are specialized areas within these fields of clinical practice, and both disciplines for NICU patients have been acknowledged since the early 1980s [9]. Neonatal therapists are in a unique position to support parent mental health in the NICU because common approaches to treatment combine complementary and alternative interventions as well as family-centered instruction [10]. Neonatal therapists are skilled in recognizing and responding to infant behavioral cues in a manner that protects the infant’s under-developed neurological and sensory systems [11]. Therapists also deliver motor interventions in the NICU that lead to short-term motor gains in preterm infant; when therapists teach parents to deliver motor interventions, improvements are seen in both motor and cognitive outcomes over the first year of life [12]. As parents practice these interventions with their infant, the infant develops muscles that support postural control and endurance while the parent gains comfort holding and interacting with their preterm infant [13]. Therefore, the therapist’s ongoing presence from birth up to hospital discharge promotes parental engagement by teaching the parent how to respond to infant behavioral cues and facilitate developmental techniques [14]. In this way, the therapist not only plays a critical role in the early social and motor development of the preterm infant, but may also support parent mental health through fostering parent competence. 

Therapist Education and Massage for Parent–Infant Outcomes (TEMPO) is a therapist-led program that uses evidence-based therapy and massage interventions facilitated between the mother and infant to support maternal mental health outcomes. TEMPO standardizes the nature and frequency of parent education through weekly scheduled sessions with a primary physical or occupational therapist. Using a family-centered approach to facilitate maternal–infant attachment [7], the therapist trains mothers of extremely preterm infants to deliver motor and massage interventions from birth throughout hospitalization. The goal of TEMPO is to support mothers to become integral members of the care team who are empowered to regularly engage with their infant in safe, meaningful ways that could lead to improved maternal mental health [14]. The primary goal of the parent study was to establish feasibility of the TEMPO intervention based on numerous benchmarks including enrollment, retainment, data collection adherence, and intervention adherence, and secondarily, objective measures of maternal mental health and infant development—all reported in a separate manuscript.

The purpose of this qualitative study is to understand parent and therapist perceptions of feasibility, acceptability, and benefits of the TEMPO program, as implemented during the parent study. This manuscript presents the results of 19 parent interviews (76% of those who completed the intervention) at the 12 month follow-up of the TEMPO program. The dataset includes 19 transcripts (each approximately three pages long and consisting of 20 questions) from phone calls occurring at 12 months post-hospital discharge between a research assistant and a parent who participated in the TEMPO study. Each “case” in the dataset is a parent, all mothers, who participated in the TEMPO study and was available for a 12-month post-hospital discharge follow-up phone call. 

We will also present the results of a focus group of four therapists who conducted the TEMPO intervention. This dataset includes a 26 page transcript of the focus group discussion led by a research assistant who prompted the therapists with 13 questions. The objective of this study is to provide parent and therapist perceptions of the feasibility, acceptability, and perceived benefits of a therapist-led weekly parent education program for parents of extremely preterm infants in the NICU.

## 2. Materials and Methods

### 2.1. Study Design

The parent study was a prospective, single group, non-randomized trial of extremely preterm infants (<28 weeks gestation) and their parents. The study was pre-registered at ClinicalTrials.gov under the identifier: NCT04121897 in October 2019. The study was approved by the (redacted) Institutional Review Board in September 2019, and recruitment began in November 2019. 

#### 2.1.1. Setting 

Dyads were recruited in the University of North Carolina Newborn Critical Care Center where ~100 extremely preterm infants are born annually. All nurses receive introductory training in developmental care upon hire but have varying experience with the neonatal population. An interdisciplinary developmental committee for the NICU (co-chaired by a therapist and nurse) provides regular education sessions throughout the year. This committee also oversees and communicates evidence-based recommendations for positioning aid/equipment use, unit protocols for sound and light control, and family-centered care policies (e.g., skin-to-skin facilitation).

#### 2.1.2. Parent Participants

To be eligible, dyads had to consist of an infant born before 28 weeks of gestation who was less than 4 weeks of postnatal age, and the biological parents had to speak English. The only exclusion criteria were genetic/chromosomal abnormalities or low bone density (defined as detected serum alkaline phosphatase activity >800 to 1000 IU/L) [15]. 

Of the eligible dyads approached by a member of the study team (parents = 35, infants = 39), 3 parents declined participation, resulting in enrollment of 32 dyads. Within the first month of enrollment, five dyads (three parents and five infants) were transferred to outside hospitals after enrollment but prior to initiating the study intervention. After these five infants were transferred, participant screening and exclusion criteria were revised to exclude dyads whose parents were considering a transfer to another hospital. Additionally, two infants died after enrollment but prior to initiating the study intervention. Therefore, the total sample consisted of 25 parents and 27 infants who participated in at least one TEMPO intervention session. Of the 27 parent–infant dyads enrolled at baseline, one additional infant was transferred to an outside hospital, and one infant died prior to hospital discharge; therefore, 25/27 dyads in total were retained for participation in the study through hospital discharge (92.5%). Nineteen of these parents (76%) were reachable by phone and agreed to be interviewed at the 2nd follow-up when their infants were of approximately 12 months corrected age (Table 1). Dyads participated in an average of 11 therapy sessions during hospitalization, but this number ranged from 5–20 sessions based on individual infant length of stay.

Ten mothers identified as white (53%), six identified as Black (32%), and three responded “prefer not to answer” (15%). Two mothers identified as Hispanic (10%). Mothers’ ages ranged from 24–51 years with an average age of 33.5 years, and infant gestational ages ranged from 24–27 weeks with an average gestational age of 26 weeks.

#### 2.1.3. Therapist Participants

A total of five therapists (two PTs and three OTs) provided TEMPO intervention to parents. All therapists were full-time therapists within the NICU and had experience and/or advanced training in infant massage and neonatal therapy practice. Prior to study initiation, the PI provided TEMPO-specific training and orientation to written educational materials and specific time frames during the study for interventions based on infant developmental presentation and age. Upon enrollment to the study, each dyad was assigned a primary therapist (either PT or OT) who scheduled and facilitated weekly visits with the parent.

### 2.2. Intervention

Enrolled dyads received standard of care + TEMPO. Differences between standard of care and TEMPO service delivery are outlined in Table 2. 

#### 2.2.1. Standard of Care

The components of standard of therapy care included:

PT and OT Initial Assessment: Initiated within the first 72–96 h of life (if infant was medically stable) and included observation of passive and active movement, positioning assessment, infant behavioral–motor regulation and maturity, and infant response to developmental support.

PT and OT Intervention at <33 weeks Postmenstrual Age (PMA): During hospitalization, infants received PT intervention 1–2x/week and OT intervention 1–2x/week for approximately 15–30 min at a time. At <33 weeks PMA, the therapist provided developmental support, positioning consultation, and parent education as the parent is available. 

PT and OT Re-assessment: Performed between 33–34 weeks PMA if infant was physiologically stable. Re-assessment included all components of initial assessment as well as evaluation of postural control in various positions, muscle tone and flexibility, active movement against gravity, and reflex development.

PT and OT Intervention >33 weeks PMA: Infants continued to receive combined PT intervention 1–2x/week and OT intervention 1–2x/week for approximately 30 min each session. At >33 weeks PMA, the therapist provided more advanced therapeutic interventions to facilitate midline orientation, encourage active movement and postural activation, and address any muscular tightness with stretching as needed.

Discharge Education: In standard of care therapy, the PT or OT reviewed a two-page handout with the parent if they were available at the bedside during the week of discharge. If the parent was not available, then the handout was left at the bedside for parent review along with therapist contact information for questions.

<4 month and 12-month Outpatient Follow-Up Visits: The Special Infant Care Clinic is a multidisciplinary follow-up clinic that follows the neurodevelopment of preterm infants discharged from the NCCC. At these visits, scheduled based on the infant’s corrected age, the PT or OT screened infants for motor delays and addressed any safety concerns, infant changes, and/or parent questions. The Bayley Scales of Infant Development IV was conducted only at the 12-month visit.

Early Intervention Referral: Based on birth gestational age, all infants in this study qualified for Early Intervention services and received referrals at hospital discharge. Whether or not the infant received physical, occupational, speech, or developmental therapies as a result of this referral was based on community therapist evaluation and family preference.

#### 2.2.2. TEMPO Intervention 

TEMPO-specific components included the following:

Early Parent Education Session: A 30 min education session initiated within one week of parent consent. A primary therapist who was a member of the study team, educated the parent about the importance of infant positioning, the impact of prematurity on the motor and sensory systems, and how to read and respond to infant behavioral–motor cues [10] using a pamphlet with pictures to supplement the verbal education.

Weekly Parent Education Sessions: Following the early parent education session, the primary therapist held weekly parent education sessions. At least two of these scheduled sessions included infant massage instruction at the infant’s bedside. On occasions when the parent was not available for in-person sessions due to illness (e.g., COVID+) or other conflicts, then the session was facilitated over video chat using an encrypted iPad. 

Infant Massage Parent Education Sessions: Parent-administered infant massage was incorporated into the therapy plan of care when the infant could demonstrate physiologic stability and maintain a normal body temperature with reasonable support outside the incubator, usually ~33–34 weeks PMA. The therapist provided education to the parent about potential benefits of massage as well as contraindications and safety considerations for massage. The therapist taught massage over at least two education sessions using the Massage+ protocol [16] that involves 30 s of infant-directed talking, 10 min of massage to infant’s head, arms, legs, chest, and back, and rocking the infant for 5 min. Minor adaptations to the protocol were made to defer head and chest massage in the setting of nasal cannula and/or EEG leads. The therapist demonstrated massage on a doll using verbal cues and written instructions to guide the parent in administering massage on the infant. Once the therapist determined the parent’s ability to safely administer massage, parents were encouraged to practice infant massage at each bedside visit outside of TEMPO sessions. Parents were encouraged to complete full body massage (e.g., back, arms, legs, chest) based on infant tolerance as often as they visited, or daily at home. Infant tolerance to massage of each body part was assessed by the therapist and discussed with the parent for carrying out in between hospital sessions and at home.

Discharge Parent Education Session: Within the week of hospital discharge, the primary therapist facilitated a 30 min face-to-face parent education session to review infant massage and age-appropriate motor activities for home. A supplemental handout and therapist contact information was provided. Parents were encouraged to provide recommended activities daily, if possible, with their infant.

Bi-weekly emails: Parents received bi-weekly emails with motor activities and massage tips for the infant’s corrected age from hospital discharge through 12 months corrected age. 

Parent Education Post-Discharge: The PI called the parent within 2 months of discharge to review the infant’s home program and massage techniques and to answer any questions. Therapist contact information and details of follow-up appointments were reviewed.

### 2.3. Outcome Measures

#### 2.3.1. Parent Interviews

The primary objective for the parent study was to establish feasibility of the TEMPO intervention through numerous targets including enrollment, recruitment, data collection, and intervention adherence. This manuscript reports the outcomes related secondary objectives of the parent study as described below in Aim 2:

Aim 2. Use results from Aim 1, including parent interview data, to refine the TEMPO intervention for future randomized controlled trials.

Please see Appendix A. for the parent interview guide.

#### 2.3.2. Therapist Focus Group

At the end of the intervention (March of 2022), the PI submitted an IRB modification and received approval to conduct a TEMPO interventionist (therapist) focus group in order to gather important data about the feasibility and acceptability of implementing the TEMPO intervention. Pleas see Appendix B. for the therapist focus group guide.

### 2.4. Data Collection

#### 2.4.1. Parent Interviews

Nineteen mothers who participated in the study intervention were interviewed over the phone by the research assistant (RA) within one week of the 2nd follow-up visit (at approximately 12 months infant corrected age) to ask for their opinions on the feasibility, acceptability, and perceived benefits of TEMPO using a semi-structured interview guide. The RA, who was not involved in the intervention sessions, elicited feedback on program materials, intervention components, and factors that influenced whether they were able to continue the protocol at various time points post-discharge. See Appendix A. for semi-structured interview questions. Each interview was audio recorded and lasted 10–20 min. The same RA that conducted all parent interviews transcribed each interview into text format. The PI checked all transcripts for accuracy.

#### 2.4.2. Therapist Focus Group

A therapist focus group was conducted over a 1 h in-person session conducted by the RA using a focus group guide (Appendix B) of 13 questions with four TEMPO interventionists (one PT and three OTs). The PI, who is a PT and also acted as an interventionist for the study, did not participate in the focus group. The session was audio-recorded and was transcribed by the same RA that conducted the focus group. The entire written transcript was checked in reference to the audio recording for accuracy by the PI.

### 2.5. Data Analysis

#### 2.5.1. Parent Interviews

Directed content analysis was used to analyze the qualitative data [17]. Because the interviews sought to answer specific questions related to the TEMPO intervention in a pragmatic way, data analysis began with use of provisional, or researcher-generated, codes and subcodes [17,18] related to three main categories: feasibility, acceptability, and perceived benefits.

All interview transcripts were reviewed by the PI and one RA who each documented memos providing insights into emerging similarities and patterns in parent responses. From these memos, the PI established six codes and eight subcodes (Table 3). The RA used these established codes and subcodes for secondary data analysis. The PI then extracted in vivo words and phrases to establish descriptive codes within each code and subcode. For example, regarding the code of “Acceptability of TEMPO”, the following in vivo words were clustered together under the descriptive code “support”: guidance, cared about me, supportive, therapist connection, one-on-one attention, by my side. The PI added the additional code of “NICU Environment” because mothers often described the emotional impact of the NICU environment without specific prompting from the researcher. 

Prolonged engagement [18] between research participants (parents) and the study team supported validity of the results. At the time of the parent interviews, the dyads had been enrolled in TEMPO for an average of 16 months, with the initial 3–4 months including weekly TEMPO sessions during hospitalization; therefore, most interviews were conducted approximately 12 months after hospital discharge. The semi-structured interview questions and pragmatic approach supported reproducibility in future research [18]. Rigor was enhanced by having the RA, who was not involved in the TEMPO intervention, conduct all parent interviews instead of the PI, who also served as an interventionist.

#### 2.5.2. Therapist Focus Group

The written focus group transcript was analyzed by two research assistants (RA) trained in qualitative methods. The transcript was initially reviewed by one RA who identified 5 codes and 16 subcodes [18] from therapists’ responses to the 13 questions used during the focus group. Using a directed content analysis approach, the RA read and created memos for emergent ideas to support identified codes and subcodes. These memos were then organized into a data matrix by code and subcode for further analysis. A second RA reviewed the transcript and made memos and then organized them into the data matrix based on the codes and subcodes defined by the first RA. “In vivo” phrases were pulled from the data matrix to provide examples for subcodes. The PI reviewed the data matrix for congruence between the two RAs (Table 4). 

## 3. Results

In the following sections, codes and subcodes will be italicized for the reader to observe patterns between parent and therapist responses. Overall, both parents and therapists viewed the TEMPO program positively. Parents and therapists shared many similar views about the program. Both parents and therapists enjoyed the focus on parent education and engagement and how that aspect of the TEMPO intervention increased parent competence and parent–infant bonding and provided support to parents. Both groups described appreciation for building a strong parent and therapist relationship throughout the NICU stay. Both groups also had positive views about infant massage in the NICU setting and acknowledged both infant-centered benefits and parent-centered benefits.

One area where parent and therapist views did not align was regarding feasibility of TEMPO. While parents noted multiple logistical challenges to regular NICU visitation, they ultimately agreed that meeting with the therapist weekly during infant hospitalization was feasible due to work flexibility, therapist flexibility in scheduling, and residing locally with support of charitable housing opportunities; however, therapists noted that the TEMPO program required more time and effort than standard of care therapy practice, and noted that multiple institutional and system-level changes necessary to implement TEMPO as standard of care.

### 3.1. Parent Interviews

#### 3.1.1. Feasibility of TEMPO

Most parents reported that meeting with the therapist on a weekly basis was feasible because of therapist flexibility in meeting times and because of residing locally. When parents were asked if they thought it was difficult to meet the therapist weekly, all but one said “no”. A few parents mentioned barriers to TEMPO that they had to overcome or consider when visiting weekly. These included work schedule, lack of maternity leave, other children at home, and having to quarantine related to COVID+ diagnosis.

I was just able to—halfway through his stay I had to go back to work, but my job was just very, um, good about letting me go when I needed to go, and I only went back part time for a while…I, you know, if I messaged (the therapist) and said I couldn’t come the day I was supposed to be, it wasn’t an issue. If I ever had to, like, meet with (the therapist) on a day I was supposed to be at work, I could move stuff around. So, both the hospital and my job made it very easy to meet with them.

(Case 101)

Common reasons provided by mothers for missed weekly sessions were work-related, caring for other children at home, and the need to quarantine due to COVID+ exposure or diagnosis.

#### 3.1.2. Acceptability of TEMPO

Overall, parents seemed to enjoy the TEMPO program and appreciated the focus on parent education and engagement and learning how to massage their infant. Parents felt these activities gave them ways to help and respond to their infant. They expressed that having tools like massage and developmental activities gave them something to do with their infant that was mutually beneficial.

Um, I liked it, it was definitely—it was nice to be able to keep my baby calm, find something new, and to feel like I was doing something to help him. In the NICU it’s always the doctors or nurse telling you what to do, you (don’t) get a say, you don’t get to do anything, so it was something I could do as a parent to help him.

(Case 104)

I feel like especially in a NICU setting it’s just hard, especially for so long, they were just basically in a place like where we couldn’t hold (the infant) a lot or touch (the infant), so I feel like being able to maximize those times was really important, and I think that it 100% helps the babies developmentally as well, so we have nothing but great things about the whole TEMPO program.

(Case 111)

Dissatisfaction with TEMPO was rarely expressed by mothers. When prompted, only one mother shared that she wanted more information to study in between TEMPO sessions. Most parents shared that they found the written pamphlets provided during hospitalization the most helpful for their learning as compared to online resources emailed after discharge. One parent explained that she did not connect with the therapist and felt this was a very important aspect of the intervention.

I think it was just personalities, it was not like anyone said or did anything differently. I don’t know if there’s a way to like make that clear to families, when they are—if anyone seems reluctant at first of like, “Would it be helpful if a different physical therapist came or would you like to be with a different”—or even just like having a different physical therapist come without them asking for it. And just kind of giving that as an option.

(Case 109)

#### 3.1.3. Perceived Benefits of TEMPO 

Several descriptive subcodes emerged from discussing perceived benefits of TEMPO for the mothers. These included (1) parent competence, (2) support, (3) mutual benefit, and (4) gratitude. When mothers were asked if they would recommend TEMPO to other parents in the NICU, 100% said “yes”.

Honestly, we loved it so much. We just felt very supported, um, you know everyone was very engaged, everyone, you know, knew (my baby). We felt very connected to the therapist.

(Case 105)

(The therapist) was very informative… I like that it was hands on so I felt like, I did not feel like so much of an outsider, like I felt included…as opposed to like sometimes the nurses would handle her care stuff, some of the nurses like hovered and did more and did not allow me the autonomy to be hands on. I like that (therapist) sat there, and like she was showing me then she had me do it, so that I felt I could independently care for my little preemie. 

Mothers also described how the therapy sessions helped them overcome fear and uncertainty related to their infant’s size and acuity.

Pretty much, um, whenever she was born, she was born 25 weeks so whenever the TEMPO therapists came to me saying that they were going to guide me through everything, ‘cause I was scared myself to even hold her, but they got me through it’.

(Case 114)

#### 3.1.4. Feasibility of Massage at Home 

Mothers who reported regularly administering massage to their infants at home often did so as part of a bedtime routine. Many mothers used massage strokes to apply lotion after bath to calm down the infant for transition to a sleepy state.

(my baby has) kind of gotten, you know, used to the routine where we give him a bath and then, you know, we grab some of the lotion and then we massage him and then put the pajama on, so I think he kind of, you know, anticipates what’s happening next, which is really cute.

(Case 119)

The majority of mothers described that it became more and more challenging to administer massage as their infant became older and more mobile, usually around 6 months of age; however, all but one mother reported that they continued performed massage at least weekly with their infant. Another stated barrier to implementing massage at home was finding the time to do it while juggling work and other childcare demands.

Um, the only thing that I could say is that the massage we haven’t been doing that since (the baby) was about six months old because she just (won’t) stay still long enough now…. She’s like ‘I don’t want it’. (laughs)

(Case 110)

#### 3.1.5. Perceived Maternal Benefits of Massage 

Overall, parents described that administering massage to their infant made them feel happy because they were doing something beneficial for their baby, that they felt relaxed (in stark contrast emotions elicited by the NICU setting), and that it afforded them time for parent–infant bonding.

It’s very calming and soothing for me or her dad, whichever one of us is doing it. Um, you know, as long as we kind of are in a situation where we can focus on her. But it’s nice because we kind of put our phones down, and you know, we’re not distracted, so I would say, it has to equally a calming and soothing effect on both of us.

(Case 105)

It’s relaxing for me as well especially if I know it’s relaxing for her and calms her.

(Case 121)

#### 3.1.6. Perceived Infant Benefits of Massage 

Interestingly, the same three descriptive codes emerged from the mothers’ perceived benefits of massage for their infants. Mothers described their infants as happy and relaxed, and appreciated more opportunity for parent–infant bonding—all previously described as benefits for the parent. In addition, a few mothers discussed how their older infants responded to massage at home through vocalizations (e.g., giggles, squeals) and physical changes such as improvements in muscle tone.

Oh, it was great, cause I mean, I’m gonna say it was great because, in my mind, I thought that was really healthy [for] her.

(Case 126)

Just how to interact with her, you know, just introducing those, uh, massage techniques and how to keep her from being nervous or, you know, just try to calm her down. It helped me try to calm down too. It helped us with the bonding.

(Case 103)

#### 3.1.7. NICU Environment

Finally, the researchers added the additional code of NICU Environment to the dataset to capture mothers’ reactions around the challenges they faced that were particular to the setting. Subcodes that emerged from mothers’ reflections on the NICU environment descriptively in direct contrast to NICU environment subcodes, which included stressful/anxious, limited control, and fear/uncertainty.

Yeah, it was a nice part of the day. It made the hospital stay not seem as hectic (laughs) and stressful. You know, you have that downtime once a week, it’s just kind of you and your baby bonding in a different way than how you normally would in a hospital and, um, to me it was a really relaxing part—it was something I looked forward to each week because it was working for both me and him.

(Case 101)

### 3.2. Therapist Focus Group

#### 3.2.1. Feasibility of Implementing TEMPO as Standard of Care

Subcodes derived from therapist discussion on the topic of feasibility included (1) barriers, (2) facilitators, (3) need to individualize plans of care within TEMPO, and (4) institutional and system-level changes necessary to implement TEMPO. Regarding barriers, therapists acknowledged that implementing weekly therapy sessions and massage education into each infant’s plan of care would not be feasible given current staffing structure and caseloads. Because much of the time that therapists spent coordinating parent and nursing schedules could not be accounted for within the billing system, therapists were concerned that implementing the TEMPO program for all patients would prevent them from meeting productivity requirements.

I think the other thing that ties into it a little bit, but as a new NICU therapist and just thinking about productivity and trying to make sure that I’m still meeting a level of productivity, but TEMPO, I felt like the work for TEMPO, you are not going to be able to bill for that, all of the coordination, that kind of stuff, but it still sucks up a little bit of your time and that goes back to being staffed adequately, right?

(Therapist 4)

We’re not staffed in a way that we could provide Tempo type clinical care to every baby on our caseload. Trying to manage parents’ schedules and work around and make the phone calls and then they don’t show up and there’s logistics that go into it.

(Therapist 1)

Beyond productivity requirements, therapists were distressed about how the intensity of the TEMPO program might affect their ability to care for other infants on their caseload. Because parent education sessions required additional time to schedule and facilitate, therapists reported having less time to treat other infants.

We only have a certain amount of time and like seeing our caseload was just so high sometimes that just we have to choose and we have to triage. (Therapist 3)

One of the hardest parts for me was ethically figuring this out because TEMPO takes so much time and extra effort that ultimately that’s time that is taken away from another baby that’s not TEMPO enrolled.

(Therapist 1)

Facilitators for maintaining weekly visits included setting up expectations between the therapist and the parent early during the study period and finding an efficient way to communicate with the parents. Most parents preferred to communicate via text, which was feasible for study purposes, but not for regular clinical care. 

Therapists also identified institutional and structural changes necessary implement a program like TEMPO. Because logistics and scheduling seemed to be a barrier, therapists considered the possibility of having designated staff support to assist with scheduling parent appointments, much like in an outpatient therapy setting. They also recognized that additional therapists would need to be hired in order to provide this level of care to all infants in the NICU.

While therapists considered TEMPO to be best practice for the extremely preterm infants enrolled in the study, therapists recognized a need to individualize plans of care within TEMPO. Therapists did not feel the program would be appropriate for all infants who receive PT or OT services in the NICU based on infant acuity level and parent availability. Therapists also felt that while the TEMPO program was of great benefit to most families and that individualizing the frequency of parent–therapist contact might be more appropriate in situations when the infant experiences prolonged hospitalization and/or medical setbacks—providing the therapist more autonomy over which patients are appropriate for weekly parent education sessions.

I think there are certain cases where it wouldn’t be appropriate for sure. For example, there’s some babies (who) are not going to survive, but we’re going to support them…also just some of our more severe diagnoses where (patients) are a lot more involved, I don’t think it would be appropriate for those either, or not all components of it anyway.

(Therapist 2)

#### 3.2.2. Acceptability of TEMPO: Clinical Best Practices

Five subcodes for the Acceptability of TEMPO related to emerging clinical best practices: (1) parent engagement and education, (2) parent and therapist relationship, (3) structure for parent education, (4) enhancement of clinical practice, and (5) detractions from clinical practice. Overall, therapists generally liked the TEMPO program and considered the components of the program, especially the focus on parent education and engagement, to be best practice. In particular, therapists recognized that impact of their own therapeutic interventions for the infant are multiplied when parents can carry out these activities between therapy sessions. Therapists believed that the weekly therapy session increased parent comfort while handling their infant and that contributed to development of parent competence. While openly acknowledging the potential for bias because of study participation, therapists also believed that infants participating in the TEMPO study had better state regulation and motor outcomes at the end of the study period than infants who had not participated in the study.

I feel like I…it’s totally just my opinion, but I do think our babies (who participated in TEMPO) with extreme prematurity have done extremely well. Not to say that there aren’t some of them who have had delays, but I’m just thinking of several, had lots of bumps along the way, and did really well…that was so gratifying to see the value. I think the difference is the family’s involvement, that they felt comfortable as caregivers to support their baby’s development. And I think that makes a huge difference. And that was so valuable for me to be able to see, for me to perceive better outcomes from that.

(Therapist 1)

Therapists also expressed that they enjoyed building deeper parent and therapist relationships throughout the NICU stay and noted that many families had made efforts to keep the therapists updated about their infant’s progress after hospital discharge. This post-hospital discharge connection also allowed the therapists to be a resource for families if needed.

Yeah, so they send me pictures and videos, and it’s the most lovely thing because…it’s good for me and my professional well-being to see where they end up, but also for them to have a point of reference if they had a question.

(Therapist 1)

The therapists, especially those with less experience, also appreciated having structure for parent education sessions. They felt that the structure of the TEMPO program motivated them to reach out to parents at a higher frequency than they would as part of standard of care.

It gave me a structure for when to introduce different topics and how to communicate with parents. That really helped me become a better therapist just by having some accountability for implementing those things.

(Therapist 4)

It definitely made me more intentional about scheduling with parents. I always, before Tempo, was hoping that I’d find a parent at a bedside, but we need to be a little bit more proactive than just hoping that they’re going to be there. That carried over to other patients as well.

(Therapist 2)

Therapists felt that there were several ways of providing the TEMPO intervention led to enhancement of clinical practice by increasing expectations beyond the standard of care.

It’s a great standard to set, to have these weekly sessions with parents…I think is very effective and it made me feel like a better clinician. I think parents felt more bonded and connected with their babies.

(Therapist 2)

However, despite positive feedback about the program, therapists also acknowledged ways that TEMPO led to detractions from best practice—especially when considering that not all infants in the NICU could be enrolled in the study. Many of the feasibility barriers previously discussed contributed to this tension between providing the best care for some infants and less-than-optimal care for others.

One of the hardest parts for me was ethically figuring this out because TEMPO takes so much time and extra effort that ultimately that’s time that is taken away from another baby that’s not TEMPO enrolled, right?

(Therapist 1)

#### 3.2.3. Infant Massage 

Therapists reported that use of infant massage in their clinical practice was an evidence-based intervention that provided a multitude of positive outcomes: (1) parent–infant bonding, (2) infant-centered benefits, and (3) parent-centered benefits. Some discussed the need to support and enhance parent–infant bonding in the NICU environment, and that infant massage was a hands-on intervention that could facilitate this well.

There’s a lot of research that says that bonding with their baby is so important for outcomes as well. Many types of outcomes. And so, if we can help facilitate that bonding and their role as a caregiver from the outset, then we’re setting them up for more success in the future.

(Therapist 1)

Therapists also acknowledged benefits solely for the infant and mother. For infant-centered benefits, physiological and medical outcomes such as improved weight gain, decreased length of stay, better respiratory effort, and improved motor patterns were discussed. For parent-centered benefits, therapists discussed the importance of giving mothers practical interventions to use with their infants and providing sense of control and knowledge that they are helping their infant. Therapists felt these parent-centered benefits ultimately contribute to reduced parent stress.

I think that’s about how I say it when I talk to parents about (massage). “This is your chance to give them this positive tactile experience”. That gives parents a sense of control, a sense of involvement. They’re really doing something positive as a caregiver for their baby.

(Therapist 2)

#### 3.2.4. TEMPO Focus Areas

Because TEMPO is a multi-component intervention comprised of (1) parent education and engagement and (2) infant massage, we sought feedback from therapists about which components of the intervention seemed most helpful or effective in their experience implementing TEMPO. Therapists all agreed that parent education and engagement was the most important, or foundational, component of the intervention. Establishing rapport and involving parents early in the hospital stay afforded more opportunities to apply targeted interventions, including infant massage.

I think the…parent education is sort of foundational for all the other things to happen…we’re only there a snapshot of time every week with these babies, but parents are the consistent caregiver. So, if we can teach them different techniques, like massage or ways to support sensory motor development, then that just makes it all work.

(Therapist 3)

Ultimately, therapists described that clinical best practice integrates multiple components of interventions, all with a different potential focus; therefore, comprehensive care of the parent–infant dyad in the NICU would include both parent education and engagement as well as infant massage.

…you can do them all in isolation of course…but in order to get the full benefit of those therapeutic interventions and our services, it’s best practice for us to integrate them together.

(Therapist 1)

#### 3.2.5. Accessibility

While the topic of accessibility was not specifically addressed in the focus group guide questions, the therapists brought up health equity-related concerns about how to provide comprehensive programs to families with limited resources and lower visitation rates. Therapists expressed frustration about not being able to address some of the resource-related challenges expressed by parents in the study such as finding childcare for the infant’s siblings at home, getting time off work, and the cost of gas. Therapists had to find a balance between encouraging parents to come for weekly visits and acknowledging logistical challenges they faced.

…families that have fewer resources and are strapped to keep their job and have all these other stressors at home…It’s much more difficult to help to support them and meet them face to face.

(Therapist 1)

Therapists felt that use of technology might help alleviate some of the challenges to regular communication with parents about their infant’s progress. For example, with the exception of the infant massage session that required hands-on education, some therapy education sessions could be facilitated by video chat when the parent was unavailable during the study period. Due to restraints around billing for telehealth sessions and privacy concerns, video chats are not currently an option for therapists and parents outside of study participation.

It’s been so nice for parents to have the option to FaceTime. And that’s something that outside of TEMPO, you can’t really do (in a therapy session).

(Therapist 4)

## 4. Discussion

The TEMPO program was highly acceptable to both parents and therapists alike. Parents and therapists shared many positive aspects of the program such as the focus on parent education and engagement, ability to build the parent and therapist relationship, and acknowledging that the program offered infant-centered benefits, parent-centered benefits, and opportunities to develop parent infant bonding. Parents and therapists seemed to disagree about how feasible the program was to implement. Once enrolled in the study, parents reported feeling committed to and looking forward to weekly therapy sessions, while therapists reported feeling unable to meet the needs of all infants on their caseload while devoting additional time and effort to dyads enrolled in TEMPO.

Parents’ positive experiences with TEMPO aligns with previous research about effective interventions in the NICU to reduce parent stress. In the systematic review and meta-analysis of interventions designed to reduce parent stress and trauma in the hospital setting conducted by Sabnis et al., they identified 46 distinct interventions that fell into four broad categories including: (1) changing NICU medical care (i.e., standards for medical information delivery, room set up, visiting hours); (2) complementary/alternative interventions (i.e., developmental therapies, massage, stress-reduction techniques and guidance); (3) family-centered instruction; and (4) psychotherapy [8]. Of these interventions, complementary and alternative medicines and family-centered instruction held the most promising effects on parental distress. 

A scoping review of the benefits of maternally administered infant massage revealed that mothers may experience positive effects of reduced anxiety, stress, depressive symptoms, and improved maternal–infant interactions over short term periods of time [19]. Related findings from our recent unpublished work also suggest that on average, parents who administer infant massage to their infants experience an associated decline in salivary cortisol, a biomarker for stress. There is little agreement or consensus, however, to support the necessary frequency and duration of infant massage programs to elicit such effects, and there is little evidence to determine if maternally administered massage has long-term benefits for parents or the parent–infant relationship. While most studied infant massage protocols are delivered at high frequencies (e.g., 3x/day) over concentrated periods of time (e.g., 1 day, 1 week) [20,21], our study differed in that parents often administered massage 3–4x/week over an average of 11 weeks of hospitalization. It is possible that parents in our study perceived similar benefits at a lower frequency but longer duration of infant massage intervention.

Parents and therapists alike recognized that the structure of a program like TEMPO established accountability for regular parent visitation and education. Dubner et al. conducted a study of mothers of infants born <32 weeks gestation to determine if maternal mental health symptoms (e.g., depression, anxiety, and post-traumatic stress) 2 weeks after birth predicted the frequency, rate, or duration of the mother’s engagement in developmental care activities such as skin-to-skin, swaddled holding, touch, and massage [22]. There were no significant differences in rates, frequency, or duration of developmental care activities between the group clinically elevated mental health scores and those without clinically elevated scores [22]. Unlike previous studies that use rates of maternal visitation and physical contact as predictors of maternal mental health outcomes [6], this study demonstrates that maternal mental health early in the hospital stay may not predict the how often mothers visit or engage in physical contact with their infants [22]. Therefore, despite baseline mental health challenges, mothers may be taught to administer developmental activities that, in turn, result in increased visitation and contact with their infant.

TEMPO may also lead to improved maternal mental health because it affords parents the opportunity to provide specialized care to their hospitalized infant in an environment that otherwise presents barriers to natural parenting practices. In a systematic review by Wang et al., “barriers to parenting” was one of the most common themes derived from multiple qualitative studies of maternal emotions in the NICU [23]. Matricardi et al. developed an intervention called “joint observation” that aimed to increase the parent’s ability to observe the infant and improve physical contact between parent and infant with the support of a physical therapist [24]. In alignment with our findings from this qualitative study, they found that this intervention reduced maternal stress—especially stress related to the marginalization of their role as a parent [24]. 

An important aspect of our findings is the marked difference in described maternal emotions elicited by the NICU environment as compared to participation in the TEMPO program. Maternal descriptions of emotions provoked in the NICU environment included uncertainty, fear, anxiety, and loss of control, which are well aligned with previous findings from both qualitative [23,25] and quantitative [24,26] studies examining parent perceptions of the NICU. In contrast, parents described the therapists implementing TEMPO as supportive and caring, and they used phrases like “reassured” and “felt comfortable” to express their emotions during TEMPO activities. It is possible that these activities helped to establish trust between therapist and parent, contributing to their ongoing positive relationship. When considering one parent’s report of difficulty connecting with their primary TEMPO therapist, it is important to consider how nonverbal communication may have been interpreted by individual parents and contributed to their learning environment [27]. For example, a parent stated that the educational content was similar when she compared two different therapists, but that she felt a mismatch of “personalities” between herself and the assigned TEMPO therapist. Therefore, it is crucial for the neonatal therapist to have good self-awareness regarding communication and their approach to education. In regard to the act of massage, parents described their own experience of administering infant massage as “soothing” and “less stressful” than other experiences in the NICU environment.

The discordant findings between parents and therapists regarding the feasibility of implementing the TEMPO program was not expected. Based on data suggesting that parents of infants in the NICU visit their infants on average between 1 to 4 days per week [28,29], we expected our parents to have more difficulty meeting therapists on a weekly basis. Overall, 87.8% of weekly planned parent education sessions took place during the study; however, this did not account for any potential rescheduling to accommodate changes in parent schedules. Parents consistently noted that therapist flexibility made weekly visits more feasible, and therapists acknowledged that the need to frequently change previously scheduled sessions to accommodate parent schedules presented significant challenges to seeing other infants outside of the study. 

While there are no standards or recommendations for frequency or duration of therapy intervention, evidence suggests that most neonatal therapists, including the therapists in this study, treat infants on their caseload 1–2 times per week on average, and this frequency may increase or decrease based on length of hospitalization, infant medical acuity, and complications of prematurity [30,31,32]. Pineda et al. conducted a survey-based study published in 2021 that revealed an average of 17 beds per neonatal full-time therapist determined that this ratio was a common indicator of adequate therapy coverage within a NICU [27]; however, some recommendations that consider the additional non-billable time necessary in neonatal PT and OT practice consider 13–15 infants per FTE more appropriate [30]. At the time of this study, our NICU had the equivalent of 2.25 full-time OTs and 2.25 full-time PTs in a 65-bed unit. Due to high levels of infant acuity in our level IV NICU, more than 90% of infants admitted received PT and OT. Our average staffing ratio of 13–14 beds per full-time neonatal therapist, despite being well-staffed compared to the national average, still presented challenges to therapists planning weekly parent education sessions with a subset of infants/dyads on their caseload. 

Therapists reported that they enjoyed providing the level of care that the TEMPO program offered. They noted many structural and institutional barriers to weekly parent education sessions including number of infants on their caseload, inability to bill for scheduling coordination, and limits to providing therapy care between nursing care times. One example of an institutional barrier in our NICU concerns timing of nursing assessments. Infant care times/nursing assessments are usually completed 3–4 h apart within consistent hourly time frames [33]. For example, the majority of infant care times during a therapist’s 8 h workday are 8:00 a.m., 11:00 a.m., and 2:00 p.m. Therapists may coordinate with the nurse to treat the infant before, during, or after the infant’s care time based on infant age, feeding requirements, and medical complexity. Therefore, most therapy treatment sessions happen in the half hour before or after these care times, providing short windows of time for productive treatment and evaluation. Adjusting nursing care times to a more staggered schedule could accommodate more therapeutic treatment sessions within an 8 h workday.

Therapists also expressed the need for additional staffing, both for more therapists to provide interventions services at recommended frequencies and also for dedicated staff members to assist with logistics of scheduling sessions with parents. This staffing service, usually exclusive to outpatient therapy environments, would require a substantial institutional and structural shift in hiring expectations and would present financial challenges due to different billing systems and reimbursement rates in inpatient and outpatient clinical settings [34].

Ultimately, therapists did not see infants at a higher frequency than standard of care practice, but they enjoyed providing more intensive therapeutic care with parents through the TEMPO intervention. In alignment with best practice in clinical care [12,13,14,35], TEMPO provided the structure to ensure that parents were delivering recommended interventions at each stage of the infant’s development. Therapists felt that they were optimizing what they could offer the infant through regular interactions with parents. Therapists were able to identify numerous evidence-based benefits of massage for both mother and infant [19,20,21] and enjoyed introducing and incorporating the massage modality as a standard part of the infant’s plan of care.

This study has limitations. Because we did not have 100% participation in parent interviews, we cannot be certain that these results collectively reflect all parent participant perspectives. Additionally, this study does not account for parents who did not originally consent to participate in TEMPO and might represent different views about weekly therapy visits during infant hospitalization. Although therapists brought up potential challenges to TEMPO when considering family social determinants of health, no parents discussed resource-related challenges during the parent interview. Further examination of parent and family characteristics is necessary to understand the generalizability of TEMPO.

## 5. Conclusions

In conclusion, both parents and therapists had positive views of a weekly parent-centered, therapist-led neonatal therapy program designed to support maternal mental health. Parents did not find weekly parent education sessions with the therapist to be burdensome or difficult to manage, largely due to therapist and personal flexibility; however, therapists did not feel the TEMPO program was feasible to implement given current standards of clinical practice, workload expectations, and staffing structure. Further study in randomized, larger cohorts is necessary to determine effects of TEMPO on objective measures of maternal mental health and infant development. Future research should also examine the cost–benefit analysis of expanding therapy services to support maternal mental health in the NICU setting, as well as the effects of the TEMPO program on parent visitation and engagement.

## Figures and Tables

**Table 1:** Parent characteristics.

Case Number	Role	Age (Years)	Race	Ethnicity	Gestational Age (Weeks)	Therapy Services after Discharge	Parent Performs Therapy Activities	Massage at 12 Months	Body Part	Difficult to Meet for TEMPO	Would Recommend TEMPO?
101_0	Mother	24	W	NH	27	Y (2x/week)	Y	Y (EOD)	Arms, legs	N	Y
102_0	Mother	U	W	NH	27	N	N	Y (EOD)	Back, legs	N	Y
103_0	Mother	40	B	NH	25	Y (1x/week)	Y (3x/week)	Y (3x/week)	Legs, feet	N	Y
104_0	Mother	25	W	NH	24	Y (1x/week)	Daily	Y (Daily)	Legs	N	Y
105_0	Mother	32	W	NH	26	Y (monthly video)	Y (3–4x/week)	Y (2–3x/week)	Back, arms	N	Y
109_0	Mother	32	W	NH	25	N	N (normal development)	Y (EOD)	Legs, feet	N	Y
110_0	Mother	34	W	NH	25	Y (2x/week)	Y (Daily)	N (stopped at 6 months)		N	Y
111_1 and 111_2	Mother	31	W	NH	26	Y (1x/week)	Y (Daily)	Y (1x/week)	Legs	N	Y
112_0	Mother	29	B	NH	27	Y (1x/week)	Y	Y (Daily)	Whole body	N	Y
113_0	Mother	37	W	NH	24	Y (1x/week)	Y (Daily)	Y (as needed)	Legs, feet	N	Y
114_0	Mother	25	U	H	25	N	Y (Daily)	Y (3–4x/week)	Nack, chest, feet	N	Y
119_0	Mother	51	W	H	27	N	Y (Daily)	Y (5–6x/week)	Back, arms, legs, chest	N	Y
120_0	Mother	39	B	NH	27	N	Y (Daily)	Y (Daily)	Legs, back	N	Y
121_0	Mother	24	B	NH	27	N	Y (Daily)	Y (Daily)		N	Y
125_0	Mother	36	B	NH	27	Y (play therapy)	Y (Daily)	Y (Daily)	Feet, hands	N	Y
126_0	Mother	40	U	NH	27	Y (1x/week)	Y (Daily)	Y (Daily)	Head	N	Y
127_0	Mother	U	W	NH	25	Y (1x/week)	Y (Daily)	Y (2x/month)	Feet, hands	N	Y
128_0	Mother	38	U	U	27	N	N	Y (Daily)	Arms, shoulders	N	Y
130_1 and 130_2	Mother	33	B	NH	27	Y	Y (Daily)	Y (3–4x/week)	Back, legs	Sometimes	Y

Key: W = White, B = Black, H = Hispanic or Latino, NH = Non-Hispanic or Latino, U = Unknown, Y = Yes, N = No, EOD = Every other day.

**Table 2:** Differences between TEMPO and standard of care intervention components.

Components of Intervention	Standard of Care	TEMPO
PT and OT Initial Assessment	X	X
Early Parent Education Session		X
Weekly PT and OT Intervention	X	X
Weekly Parent Education Sessions		X
Infant Massage Parent Education Session		X
Discharge Handout	X	X
Discharge Parent Education Session		X

**Table 3:** Parent intervention codes and subcodes.

Code	Subcode	Definition	Descriptive Codes	In Vivo Phrases Examples
Feasibility of TEMPO	Facilitators of TEMPO	Apply this code when text describes situations or factors that made weekly participation in TEMPO feasible, easier, or more reasonable.	Work flexibility	“part-time” “flexible schedule”
			Therapist flexibility	“able to reschedule” “communication”
			Residing locally	“staying at Ronald McDonald House”
			Use of technology	“able to…facetime me if I could not physically be there”
	Barriers to TEMPO	Apply this code when text describes situations or factors that made weekly participation in TEMPO more difficult or challenging.	Work-related	“could only visit certain times” “no maternity leave”
			Other children at home	“we also had a 3 year old at home”
			COVID+	“had to quarantine”
Feasibility of Massage at Home	Facilitators to Massage at Home	Apply this code when text describes situations or factors that made parent-administered massage feasible, easier, or more reasonable	Part of bedtime routine	“something we do before he goes to bed”
			Use to calm down the infant	“it kind of, like destimulates her”
	Barriers to Massage at Home	Apply this code when text describes situations or factors that made parent-administered massage more difficult or challenging.	Infant older and more mobile	“bigger” “squirmy” “mobile” “rolling over”
			Time	“finding the time” “working full time”
Acceptability of TEMPO	Likes	Apply this code when text describes components of the TEMPO program that mothers liked or enjoyed.	Written information	“helpful in learning about like what to expect”
			Learning how to massage	“helpful” “enjoyable”
			Parent Education and Engagement	“learning ways to help” “knowing what to do”
			Set aside time	“looking forward to”
			Parent and Therapist Relationship	“one on one” “connection”
	Dislikes	Apply this code when text describes components of TEMPO program that mothers did not like or enjoy.	Wanted more information	“would have loved to have more things written down to look over”
			Did not connect with therapist	“connection between PT and parents is important”
Perceived benefits of TEMPO		Apply this code when text describes ways that the mother felt that the TEMPO program was beneficial or helpful	Parenting competence—parent’s ability to care for infant improves	“reassured” “prepared” “felt comfortable”
			Mutual benefit—benefits both mother and infant	“working for both me and him” “helps baby as well”
			Support—parent felt supported by therapist	“guidance” “cared about me” “by my side”
			Gratitude—parent felt sense of gratitude for therapist	“appreciated” “sad it’s over” “glad we did it”
Perceived benefits of Massage	Infant Benefits	Apply this code when text describes ways that the mother felt that parent-administered massage was beneficial or helpful for the infant.	Happy	“excited” “enjoys”
			Increased vocalizations	“giggles” “laugh”
			Relaxed	“calm” “relaxed” “still”
			Parent–infant bonding	“focus” “stare”
			Physical changes	“muscles relax”
	Parent Benefits	Apply this code when text describes ways that the mother felt that the parent-administered massage was beneficial or helpful for the mother.	Happy	“great” “happy”
			Relaxed	“helps calm” “soothing” “less stressed”
			Improved mental health	“less stressful” “less worried”
			Parent–infant bonding	“memory” “focus”
NICU Environment		Apply this code with text describes ways that the NICU environment influenced the mother’s emotional state	Stressful/anxious	“hospital stay… hectic”
			Limited control	More in control
			Fear/uncertainty	‘cause I was scared”

**Table 4:** Therapist focus group codes and subcodes.

Code	Definition	Subcode	Definition	In vivo Phrases Examples
Feasibility of Implementing TEMPO as Standard of Care	Apply this code when therapists discuss barriers and facilitators around TEMPO becoming part of standard of care for the Newborn Critical Care Center, if it is appropriate for all infant populations, and necessary changes to make TEMPO implementable.	Barriers	Apply this code when text describes barriers to TEMPO as Standard of Care	“you are not going to be able to bill for…all of the coordination, that kind of stuff, but it still sucks up a little bit of your time”
		Facilitators	Apply this code when text describes facilitators to TEMPO as Standard of Care	“…we (parent and therapist) had this mutual understanding that we’re going to do this thing together and I think it set us up for more success because of that.”
		Needs to individualize plans of care within TEMPO program	Apply this code when text describes certain populations that would benefit most from TEMPO and/or need for individualized plan of care within the TEMPO framework	“So maybe having a best practice, but also off ramps that you could go depending on the patient.” “…there are certain cases where it wouldn’t be appropriate for sure”
		Institutional and System Level Changes necessary to implement TEMPO	Apply this code when suggested changes are at the systems or organizational level in order to implement TEMPO	“This is best practice, it really is. But logistically, is this where we can go?” …”we only have a certain amount of time and like seeing our caseload was just so high sometimes that we have to choose and we have to triage.”
Acceptability of TEMPO: Clinical Best Practices	Apply this code when text describes the best practice for the NICU clinical setting for physical therapists or what best practice would look like in an ideal setting.	Parent Education and Engagement	Apply this code when text describes the role or importance of parent involvement in clinical best practices for NICU therapists	“(parents) felt comfortable as caregivers to support their baby’s development…that makes a huge difference. And that was so valuable for me to…perceive better (infant) outcomes” “therapists come and go but parents are the constants”
		Parent and Therapist Relationship	Apply this code with text describes therapists liking increased time with parents	“relationships with family is the biggest component that I enjoyed”
		Enhancement of Clinical Practice	Apply this code when text describes how TEMPO enhanced or changed clinical practice	“If we were implementing best practice, that’s what it would look like.” “made me feel like a better clinician”
		Structure for Parent Education	Apply this code when text describes therapists liking structure and motivation to connect with parents	“made me more intentional about scheduling with parents” “…having the structure laid out in that accountability, I really appreciated that.” “TEMPO gave me a structure for when to introduce different topics and how to communicate with parents.”
		Detractions from Best Practice	Apply this code when text describes how TEMPO detracted from best practice	“(TEMPO) might affect other babies because I might not have enough time to see two babies this care time.” “One of the hardest parts for me was ethically figuring this out because TEMPO takes so much time and extra effort that ultimately that’s time that is taken away from another baby that’s not TEMPO enrolled, right?”
Infant Massage Benefits	Apply this code when text describes the usefulness or benefits of infant massage, and/or why it is used or indicated	Parent–infant Bonding	Apply this code when benefits of massage are referencing parent–infant interaction, bonding or attachment	“infant parent bonding is obviously at the forefront’
		Infant-centered Benefits	Apply this code when text describes infant-related benefits of massage	“…a lot of literature that there’s weight gain, decreased length of stay, improved respiratory efforts, their motor patterns can be…optimized with the integration of such a positive sensory stimulation.”
		Parent-centered Benefits	Apply this code when text describes parent-related benefits of massage	“…gives parents a sense of control, a sense of involvement. They’re really doing something positive.” “Helps relax them, decrease their stress.” “…for their breast milk production, pumping.”
TEMPO focus areas	Apply this code when someone discusses their thoughts on TEMPO focus areas: 1) parent education and engagement, 2) infant massage, and thoughts on their importance or how they are addressed by therapy	Parent Education and Engagement	Apply this code when text describes parent education and engagement focus area	“Parent education is sort of foundational for all the other things to happen.”
		Multi-component intervention is optimal	Apply this code when text describes the need to integrate multiple components of interventions	“…you can do them all in isolation of course…but in order to get the full benefit of those therapeutic interventions and our services, it’s best practice for us to integrate them together.”
Accessibility	Apply this code when text describes therapist ideas or concerns about accessibility of weekly parent education	Health Equity	Apply this code when text describes therapists concerns about the accessibility of TEMPO to parents with few resources	“…families that have fewer resources and are strapped to keep their job and have all these other stressors at home…It’s much more difficult to help to support them and meet them face to face.”
		Use of Technology	Apply this code when ideas around technology were used to support parent education	“it’s been so nice for parents to have the option to FaceTime.”

## Data Availability

Data available on request.

## Appendix A. Parent Semi-Structured Interview Guide

1. Have you received our emails since your hospital discharge?
2. Is your baby currently receiving outpatient or Early Intervention physical therapy or occupational therapy?
3. Do you have any questions for me about physical therapy interventions or massage with your baby?
4. Do you have all of the materials you need to complete physical therapy interventions and massage with your baby (written instructions, massage oil, etc.)?
5. How often have you tried physical therapy interventions with your baby? Have you tried any of the activities from the email blasts or from your last clinic visit with the PT?
6. How often have you tried massage with your baby?
7. Which body part of the baby do you massage most often?
8. What barriers are there to doing massage with your baby at home now, if any?
9. How do you feel when you massage your baby?
10. How does your baby respond when he/she is massaged?
11. Did you find the information you received in the hospital from the TEMPO program helpful? What in particular do you remember or remember being helpful?
12. Were the materials you received (instruction packets) useful for learning about your infant?
13. Did you like learning to massage your baby in the hospital?
14. Was it difficult to commit to meeting with the therapist for the TEMPO program weekly?
15. If you tried the video chat option for the weekly TEMPO program, did you find it helpful?
16. Did you like receiving the email blasts? Did you find that you received too many or not enough? Was the information relevant or helpful? Did the emails help you remember to try some activities or massage?
17. Was the phone call before first follow-up appointment helpful for you? Did you find that you were able to keep up physical therapy interventions and massage more often at home after the phone call?
18. Was the massage review at the first follow-up appointment helpful for you? Did you find that you were able to keep up massage more often at home after the review?
19. Overall, what did you think of the TEMPO program?
20. If you had a friend with an extremely preterm infant, would you recommend they participate in the TEMPO program?

## Appendix B. Therapist Focus Group Guide

Thinking about your position as a neonatal therapist…

1. What is the role of parent involvement in clinical best practices for neonatal therapists?
2. Can you describe best practice for the NICU clinical setting for therapists?
3. What would best practice look like in an ideal setting?
4. In what ways did participating in TEMPO enhance or changed your clinical practice, if at all?
5. In what ways did participating in TEMPO detract from your clinical practice, if at all?
6. What is the usefulness of infant massage in the NICU setting? What do you use it for? Why is it indicated?
7. TEMPO focuses on 2 main areas: (1) parent education and involvement; (2) infant massage. Do you feel that any one of these areas is more important than another? Are they all equal in how they should be addressed by therapy?
8. What were the challenges to implementing weekly therapy sessions with parents?
9. What made it easier to implement weekly therapy sessions with parents?
10. What would make it easier? What could change?
11. Were the written materials helpful for implementing TEMPO? Specifically ask about:
12. Early education packet, discharge packet, and massage instructions
13. Is there additional information you would include or remove?
14. What did you generally like about TEMPO?
15. What did you generally not like about TEMPO?
16. If you could change anything(s) about TEMPO, what would it(they) be?
17. How would you feel about TEMPO becoming part of standard of care for the NICU in the future? Is it appropriate for all infants, diagnoses, etc.? Or more appropriate for certain populations?
